# Carry-Over or Compensation? The Impact of Winter Harshness and Post-Winter Body Condition on Spring-Fattening in a Migratory Goose Species

**DOI:** 10.1371/journal.pone.0132312

**Published:** 2015-07-02

**Authors:** Kevin K. Clausen, Jesper Madsen, Ingunn M. Tombre

**Affiliations:** 1 Aarhus University, Department of Bioscience, Grenåvej 14, 8410, Rønde, Denmark; 2 Norwegian Institute for Nature Research, Department of Arctic Ecology, Fram Center, N-9296, Tromsø, Norway; Università degli Studi di Milano-Bicocca, ITALY

## Abstract

Environmental conditions at one point of the annual cycle of migratory species may lead to cross-seasonal effects affecting fitness in subsequent seasons. Based on a long-term mark-resighting dataset and scoring of body condition in an arctic breeding goose species, we demonstrate a substantial effect of winter harshness on post-winter body condition. However, this effect was compensated along the spring migration corridor, and did not persist long enough to influence future reproduction. This highlights the importance of temporal scale when assessing impacts of environmental effects, and suggests a state-dependent physiological mechanism adjusting energy accumulation according to internal energy stores carried into spring. In support of these findings, the development of body condition was unaffected by whether geese used supplementary feeding sites or not. While there was no effect of winter harshness on the average population pre-breeding body condition, individual variations in early spring body condition (probably related to different life-histories) were partly traceable throughout spring. This strongly indicates a carry-over effect on the individual level, possibly related to differences in dominance, site use, disturbance or migration strategy, which may potentially affect future reproduction.

## Introduction

The relative stability of weather forces, often referred to as a region’s climate, is what define the world’s biomes, and hence a main driver of animal abundance and distribution [1,2]. In addition, seasonal weather patterns, characterised as predictable changes during an annual cycle, give rise to large temporal variation in primary production affecting all higher trophic levels. The associated seasonal fluctuations in food availability and energy demand triggers a wide array of adaptations among animals to cope with the changing conditions, which may be physiological, morphological or phenological in character [3,4,5]. To many organisms winter, with low food availability and cold temperatures, is the most challenging season for coping with environmental stress. As a consequence, winter conditions may be a bottleneck to population increase in many organisms by means of either directly increased mortality [6,7] or negative long-term effects affecting future fitness [8,9]. While an assessment of increased mortality is often rather straightforward, quantifying potential long-term effects has proven much more difficult.

Migratory waterbirds are greatly affected by weather throughout their annual cycle, and winter is one of the energetically most challenging periods to many species [7,10,11]. Post winter waterbirds refuel their energy stores to prepare for the upcoming spring migration and breeding event, often demonstrating high rates of food consumption and rapid increases in body condition [12,13]. This build-up of energy stores during spring is often referred to as spring-fattening. Most waterbird species of the western Palearctic winter in north-western Europe, and the winter conditions in these areas may vary greatly from year to year. As a consequence food availability, energy expenditure and migration distance may fluctuate among winters [14] and influence post-winter body mass and the starting point of spring-fattening. To what extent the energetic impacts of a harsh winter persists throughout spring is poorly known, and studies thoroughly addressing the potential long-term effects of winter harshness are lacking. At one extreme, cold winters might result in a persistent lower body mass up until the following breeding season, but geese may also to some extent be able to compensate the energetically lower starting point by increasing the rate of spring-fattening.

The amount of energy needed during spring-fattening probably relates to the body condition of individual birds in early spring, and individuals might adjust their energy intake rate according to the amount of reserves they carry into spring. If this is indeed the case, spring-fattening might be considered a state-dependent physiological mechanism of adaptive behaviour [15]. This would imply that the development in spring body condition is not just a product of food availability, but to a large extent driven by the internal energetic state of individual geese. Early spring body condition however, is not just a product of the preceding winter, and individual variation in internal energy stores carried into spring are likely affected by different life-history factors such as dominance rank, family status, migration history, age, illness, disturbance, etc. Irrespective of the preceding winter, the life history-driven variation in early spring body condition might induce carry-over effects in individual geese affecting future fitness. Carry-over effects are processes occurring in one season that result in different levels of condition, consequently affecting individual performance in a subsequent period of the annual cycle [16,17]. Carry-over effects from a low early spring body condition may include lower energy stores, altered migration phenology, changing immunity against pathogens and lower reproduction [8,9,18,19], which might all in turn shape the future life-history of individual organisms.

Identifying long-term impacts, state-dependent foraging and individual carry-over effects depends on the ability to 1) track individuals through time and 2) continuously measure state (body condition) to evaluate potential impacts. In this study we apply a unique 23 year data set of body condition assessments of individually marked pink-footed geese (*Anser brachyrhynchus*) to carry out two different analyses investigating these relatively undescribed features of waterbird ecology. First, we analyse the effect of winter harshness on post-winter body condition, and assess to what extent this effect persists to the late spring pre-breeding period. This analysis is carried out on a population level and aims specifically to investigate how winter temperature affects average body condition of the entire population. Secondly, we examine whether individual birds are able to compensate intraspecific differences in post-winter body condition during spring by means of state-dependent spring-fattening rates, and evaluate to what extent variation in early spring body condition carries over to the late spring period. To identify individual carry-over effects and state-dependent foraging responses, this analysis is carried out on the level of individual geese tracked through time, and while the population-level analysis is designed only to describe annual body condition in relation to harshness of the preceding winter, the individual-level approach looks at variation in body condition that may be driven by any life history-event such as differences in dominance rank, family status, site use, migration history, age, illness and disturbance. For the population analysis we defined two hypotheses stating that: 1) Average body condition at the start of spring-fattening (early spring) is related to winter temperature, and lower body condition associated with a preceding cold winter, and 2) The population is, at least partly, able to compensate the negative effect of harsh winters on early spring body condition during spring-fattening. For the individual analysis we hypothesised that: 3) The gain in body condition of individual geese during spring-fattening is state-dependent, and therefore inversely related to early spring body condition.

Since the 1970s, between one and five fields at the Danish spring staging sites of pink-footed geese have served as supplementary feeding areas to alleviate conflicts with agricultural interests [20,21]. As supplementary feeding could potentially boost the increase in body condition of geese, the fourth hypothesis was defined to test the effect on spring-fattening rates by comparing condition of geese inside and outside these areas. In accordance with the state-dependent hypothesis predicting that geese adjust their spring-fattening according to state, we hypothesized that 4) Supplementary feeding would not affect body condition.

## Methods

### Focal species

The Svalbard-breeding population of pink-footed geese winters in Denmark, The Netherlands and Belgium, where they forage on pastures, marshes and agricultural fields [22,23]. In early spring, the majority of the population congregates in western Jutland, Denmark, before gradually migrating northwards in April to stopover sites in Trøndelag (mid-Norway) and Vesterålen (north Norway). By mid-May the geese migrate from north Norway to the Svalbard breeding grounds [24]. Since 1990, the population has been subject to a long-term ringing scheme with neckbands, and more than 3700 geese have been captured and individually marked. The majority of birds were caught in the Danish spring staging sites, but additional captures have been carried out during the post-breeding moult on Svalbard.

In Denmark geese were caught by cannon-netting and transferred to a tent. Each individual was ringed and neckbanded and returned to the tent, and all geese were released simultaneously in a flock. License to ring and use neckbands, as well as ethical approval of the work, was issued by the Ringing Central of the Zoological Museum, Copenhagen, Denmark. The capture of geese was carried out on state-owned land, and the permit to catch was issued by the National Nature Agency. In Svalbard, Norway, geese were rounded up during the moult of remiges. Geese were driven into a corral and adults and young geese separated. All individuals were ringed and adult geese neckbanded and returned to the corral. When the ringing was completed, all geese were released simultaneously to ensure that they stayed together as a flock. Ringing license was granted by the Ringing Central, Stavanger Museum, Norway, and the permit to use neckbands was granted by the Norwegian Committee for Animal Welfare (Forsøksdyrudvalget). The permit to catch geese in Svalbard was issued by the Governor of Svalbard. All marking and re-sighting data have been stored at www.geese.org.

### Body condition assessments

Body condition assessments of neckbanded pink-footed geese has been conducted annually and systematically since 1990 from when geese arrive in Denmark (late winter, early spring) to departure from Vesterålen (late spring), by assessing the abdominal profile index (API, Sensu [25]) of individual birds. Based on the sagginess of their abdomen, birds are scored on a 1–7 scale following a standardised protocol [26]. Observations were carried out by trained and experienced observers who were inter-calibrated in scoring APIs. Abdominal profile indexes are widely accepted as good proxies of waterfowl body condition [27], and the API of pink-footed geese correlates nicely with both body mass, fat stores and reproductive output [26,28], confirming the validity of this index as a measure of nutritional state and ultimately fitness in this species. The use of neckbanded birds enabled us to track the body condition of individual geese through time, allowing for long-term assessments of potential carry-over effects. The presence of neckbands does not have any long-term negative effect on the geese [29]. Ringing data, re-sightings and body condition assessments of individual birds is available as online supporting information (S2 Table and S3 Table).

### The effect of winter harshness on average population body condition

As a measure of winter harshness we used an overall average winter temperature (Dec-Feb) from Esbjerg (Denmark), Leeuwarden (The Netherlands) and Oostende (Belgium), covering the three main wintering areas of this population. Temperature data were acquired from the National Climatic Data Center (NCDC) of the National Oceanic and Atmospheric Administration (NOAA, http://www.ncdc.noaa.gov). Average winter temperatures from the three sites were highly correlated (average Pearson’s r = 0.90), indicating substantial similarity of weather conditions and winter harshness among wintering sites. As a consequence we relied on the overall average winter temperature as a proxy of winter severity in near-coastal areas of north-western Europe.

We hypothesized that potential effects of winter harshness on average goose body condition should be most pronounced in early spring immediately after winter, and used the average March API of birds in Denmark as a measure of winter impacts. To investigate the temporal persistence of the impact on average body condition, the development in API were analysed separately for March (Denmark), April (Denmark and Trøndelag) and May (Denmark, Trøndelag and Vesterålen), corresponding to the time when birds reside at these staging sites. For all month and site combinations we developed a general linear mixed model with “Year” and “Bird ID” as random effects and “Day of Month”, “Winter temperature” (the abovementioned overall average) and the interaction between these as fixed effects. The random effects were included to control for the confounding effects of individual variation in body size and annual variation unrelated to winter harshness. Hence, this analysis was constructed solely to explore the effect of winter temperature on average body condition development throughout spring. “Day of month” was incorporated to control for the temporal increase in body mass during each month, which is considerable, especially in late spring [26]. Because of gender-specific development in the API of pink-footed geese [29], males and females were analysed separately. To avoid potential age-specific effects the model included only API assessments of adult birds (> 2 years old), and data collected in the year of ringing of individual birds were discarded to eliminate the effects of capture [29].

### State-dependent effects of individual differences in early spring body condition

In order to evaluate whether early spring body condition affected subsequent spring-fattening we used body conditions from all individuals with API assessments in both March, when birds reside in Denmark, and May, when geese are at their final spring staging site in Vesterålen just before migrating to the breeding grounds. This enabled us to investigate individual variation in the rate of spring-fattening, and should reveal potential state-dependent mechanisms operating on this process. Treating May APIs as a function of March APIs, a linear regression with slope 1 would imply that no compensation was present, while a slope of 0 would indicate full compensation.

Variation in early spring body condition may relate to a number of factors such as differences in dominance rank, family group size, migration strategy, site use, age, illness, disturbance and weather impacts. Clarifying which of these impacts might lead to carry-over effects on individual geese is not possible from this analysis alone, but the approach can reveal whether birds are able to compensate a lower starting point or might be restricted by their energetic state in early spring. Hence, this analysis was made to investigate the potential for state-dependent foraging and explore possible carry over effects related to the entire life history of individual geese. When more than one API assessment was available from a site the average value was used, and like above we restricted our data to include only adult birds and discarded data collected in the year of ringing.

### Supplementary feeding

Supplementary feeding with cereal grain has taken place during the study period at up to five different locations in western Jutland, Denmark, where geese gather in large flocks resulting in agricultural damage. Up to 0.5–1 tons of grain was supplied per site during late March-April (see [20] for details). The effect of supplementary feeding on goose body condition was tested using all API assessments from April (the main supplementary feeding period [20]) to compare the body condition of birds exploiting and not exploiting these areas. This was carried out using a general linear mixed model with “Year” and “Bird ID” as random effects and “Day of month”, “Sex” and “Supplementary feeding” as fixed effects. The “Supplementary feeding” variable distinguished between API assessments of birds within and outside the supplementary feeding sites. The interaction “Sex*Supplementary feeding” was included to test for potential different responses between the two sexes, and the interaction “Year*Supplementary feeding” to capture potential differences in amount and timing of cereals applied. We cannot completely rule out that geese exploiting supplementary feeding sites occasionally foraged on regular fields in the vicinity of these supplementary feeding areas, but geese scored in areas with no supplementary feeding only had access to regular fields. The comparison in this analysis is therefore between two groups of birds that either 1) used, and always had access to, fields with supplementary feeding or 2) had no access to these fields. Observations of neckbanded geese suggest that in any single year only ≈ 7% of all birds are seen inside and outside the supplementary feeding areas during the monitoring period, suggesting that this distinction is reliable to a great extent. All statistical analyses and graphical representations were completed in R 3.0.2 [30], and mixed models were fitted using the lme4 package.

## Results

### The effect of winter harshness on average population body condition

**Fig 1:**
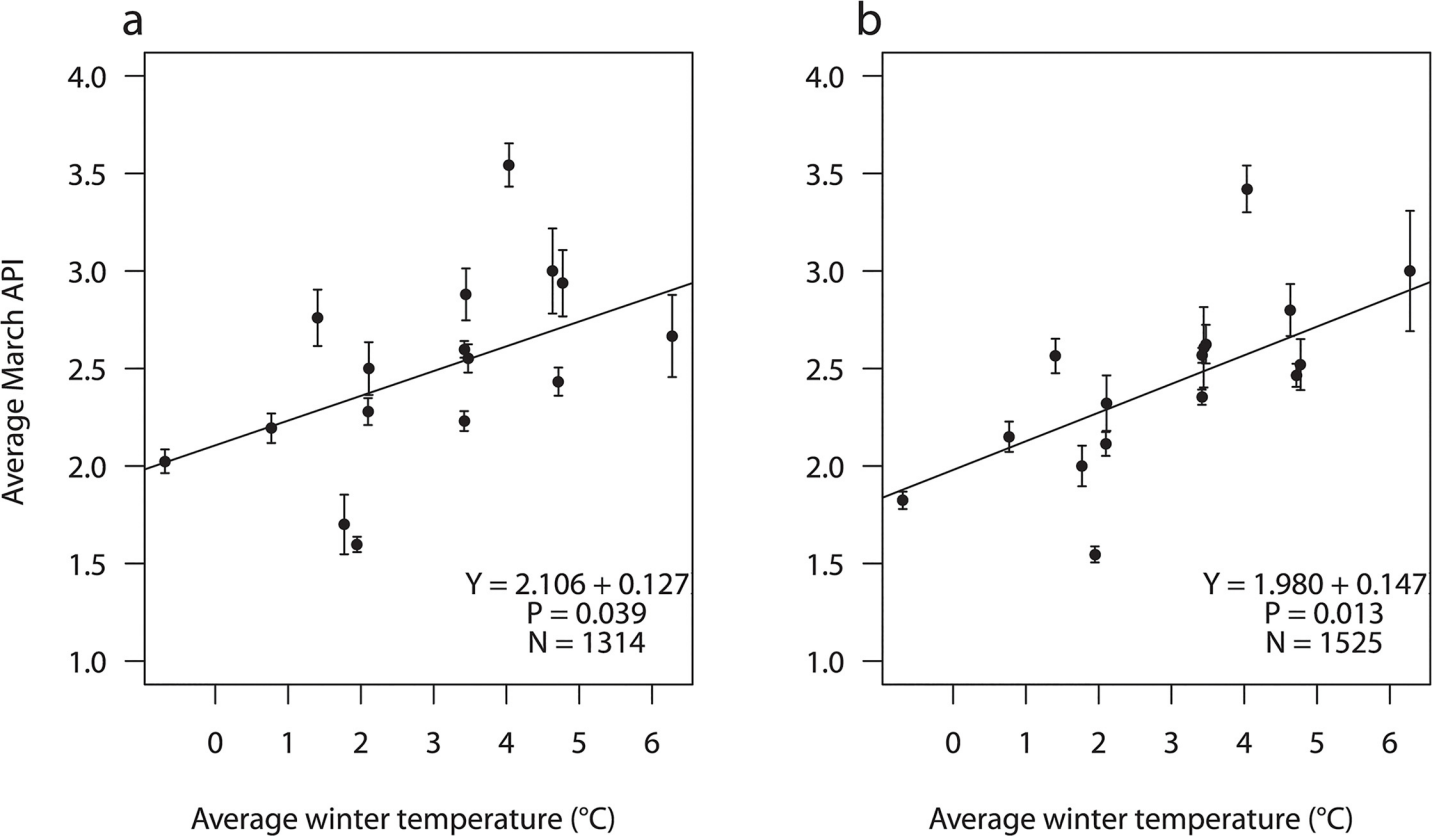
Relationship between average winter temperature and March body condition of pink-footed geese *Anser brachyrhynchus*. Average March body condition was assessed from the abdominal profile index (API) of (a) females and (b) males. Lines indicate least square fits from the linear mixed model (Table 1), and bars indicate standard errors. N indicates the number of geese included in the analysis.

During 1991–2013, average winter temperature varied between -0.67°C (1996) and 6.28°C (2007, see S1 Table). Winter harshness affected March API of both female and male pink-footed geese (Fig 1, Table 1). The effect corresponded to a drop of 0.127 and 0.147 API scores respectively for each degree drop in average winter temperature (Table 1). The effect of winter on goose body condition persisted in April among birds staying in Denmark, but was not discernible in May. Also, for both sexes there was no support for an effect of winter on API at the subsequent spring staging sites in Trøndelag and Vesterålen (Table 1). The increasing variance around the coefficients in Table 1 from March to May indicates that winter temperature became an increasingly poorer predictor of goose body condition during this period, which is what would be expected from a gradual compensation of this effect and a growing temporal and spatial separation between explanatory and dependent variables. In order to compensate the effect of cold winters on early spring body condition a significant negative interaction between “Winter temperature” and “Day of month” (indicating that rates of increase in API were steeper following cold winters) should be expected during spring-fattening. Our results indicate a significant interaction in late spring, which is in good agreement with the concurrent fading effect of winter harshness (Table 1). With ideal temporal resolution of our API data one would expect a non-significant interaction term in the days just prior to final migration (indicating that geese had reached full compensation), but in order to ensure appropriate sample sizes and acknowledge the ordinal nature of API scores we have refrained from further temporal splits of these data. As a result, there was no indication of a persistent long-term effect affecting average pre-breeding body condition of the population as a whole. As expected, day of month was significant for all site, sex and month combinations, indicating that geese build up energy stores (gradually increasing API scores) at all three staging sites on their northbound migration.

**Table 1:** Fixed effects output of the general linear mixed model to explain temporal persistence of the winter carry-over effect on pink-footed goose *Anser brachyrhynchus* body condition at three consecutive spring staging sites (Denmark, Trøndelag and Vesterålen). Presented estimates are coefficients from a model with API (abdominal profile index) as response variable, “Year” and “Bird ID” as random effects and “Day of Month”, “Winter temperature” and the interaction between these as fixed effects. Winter temperature is the average December-February temperature (°C) in the preceding winter, and N the number of geese (females & males) with abdominal profile index (API) assessments for all combinations of month and staging sites. Day of month was fitted as a continuous variable (covariate).

	Females			Males		
Denmark	Estimate	SE	P value	Estimate	SE	P value
March (N = 1314 & 1525)						
Day of month	0.031	0.002	<0.001*	0.029	0.002	<0.001*
Winter temperature	0.127	0.056	0.039*	0.147	0.052	0.013*
Day of month x Winter temperature	0.004	0.003	0.392	0.003	0.003	0.741
April (N = 3591 & 3845)						
Day of month	0.069	0.001	<0.001*	0.054	0.001	<0.001*
Winter temperature	0.102	0.046	0.048*	0.128	0.052	0.026*
Day of month x Winter temperature	-0.001	0.001	0.397	-0.000	0.001	0.985
May (N = 911 & 985)						
Day of month	0.044	0.007	<0.001*	0.019	0.007	0.011*
Winter temperature	0.095	0.080	0.268	0.088	0.133	0.527
Day of month x Winter temperature	-0.008	0.003	0.015*	-0.005	0.003	0.055
**Trøndelag, Norway**						
April (N = 707 & 655)						
Day of month	0.062	0.007	<0.001*	0.062	0.006	<0.001*
Winter temperature	0.030	0.076	0.707	0.022	0.085	0.807
Day of month x Winter temperature	0.006	0.004	0.133	0.005	0.004	0.146
May (N = 1573 & 1622)						
Day of month	0.059	0.004	<0.001*	0.031	0.004	<0.001*
Winter temperature	-0.002	0.072	0.974	-0.043	0.088	0.644
Day of month x Winter temperature	-0.013	0.002	<0.001*	-0.015	0.002	<0.001*
**Vesterålen, Norway**						
May (N = 3018 & 3102)						
Day of month	0.069	0.004	<0.001*	0.053	0.004	<0.001*
Winter temperature	0.080	0.125	0.532	0.073	0.140	0.611
Day of month x Winter temperature	-0.011	0.003	<0.001*	-0.016	0.003	<0.001*

* Significant effects on α-level 0.05

### State-dependent effects of individual differences in early spring body condition

**Fig 2:**
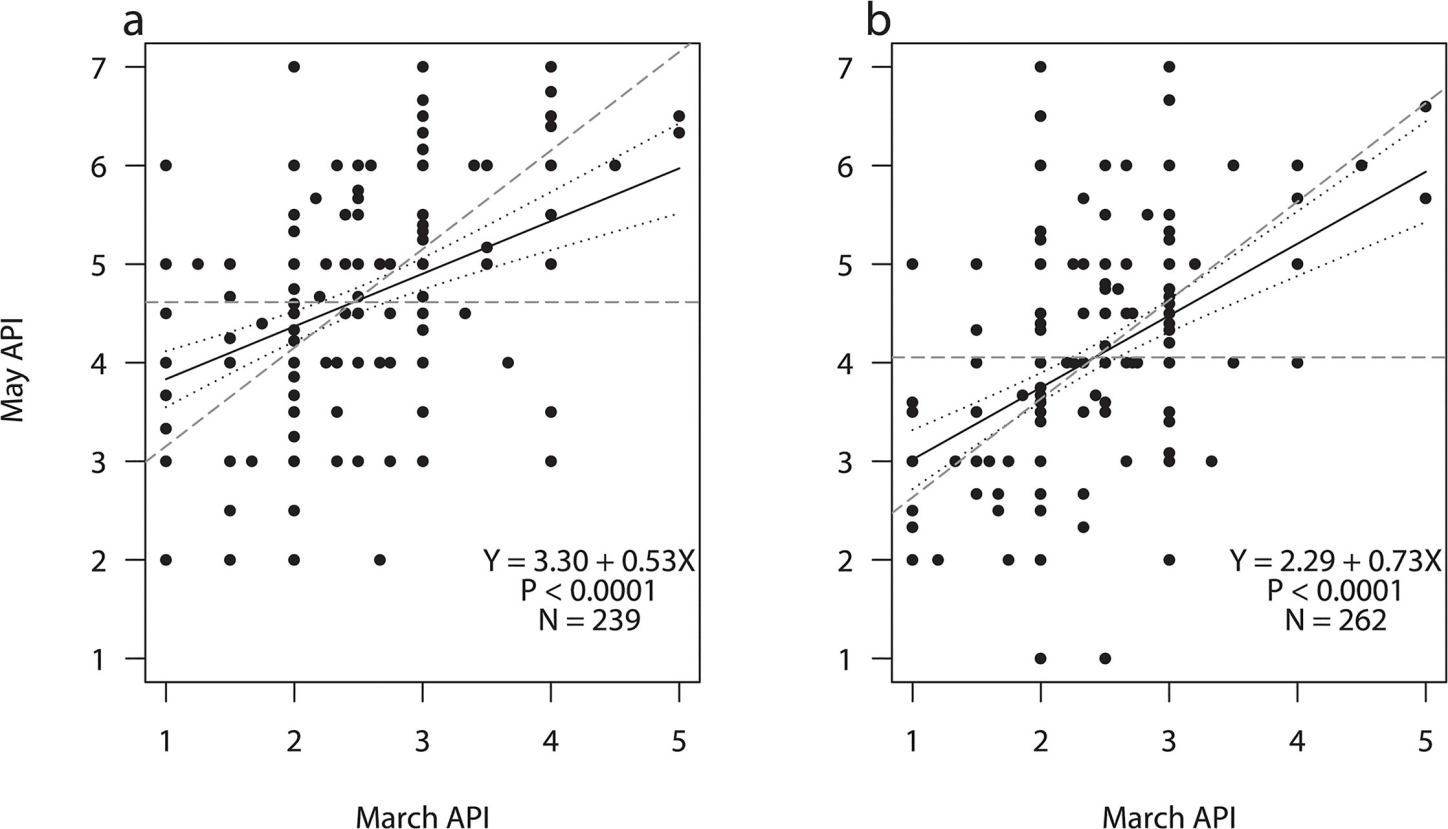
Relationship between early (March) and late (May) spring body condition of pink-footed geese *Anser brachyrhynchus*. Average body condition was assessed from the abdominal profile index (API) of (a) females and (b) males. Black lines indicate the linear fit with 95% confidence limits, and grey dashed lines indicate hypothetical fits corresponding to no compensation (slope = 1) and full compensation (slope = 0). N indicates the number of geese included in the analysis.

For both sexes, the linear regression of late spring body condition on early spring body condition had slopes that were significantly smaller than one (no compensation, Fig 2), entailing that spring-fattening rates were inversely proportional to early spring body condition. This indicates a state-dependent energy accumulation where geese entering spring in poor condition built up body reserves to a greater extent than well-conditioned birds. However, both slopes were also significantly larger than zero (full compensation, Fig 2), suggesting that compensation was incomplete and that an individual carry-over effect of early spring body condition persisted in the subsequent period. The slope of the regression line for females (0.53±0.09) was smaller than for males (0.73±0.10), indicating that females compensated a poor early spring body condition to a greater extent than their male counterparts. This might be the result of a higher body condition target among females preparing the forthcoming breeding event, which is supported by the fact that females generally reach higher APIs than males in late spring. The amount of individual variation in March body condition was not related to winter harshness (F_1,14_ = 0.16, P = 0.692), indicating that although winter harshness influenced the average body condition of the population as a whole, individual variation was probably driven by other life history events.

### Supplementary feeding

Supplementary feeding did not seem to affect the build-up of body mass during spring-fattening. April API assessments of pink-footed geese from fields with supplementary feeding averaged 3.61 (SE = 0.02) and was identical to APIs scored outside these areas (Mean = 3.61, SE = 0.03, Table 2).

**Table 2:** Fixed effects model output of the general linear model to explain the effect of supplementary feeding on April API of pink-footed geese *Anser brachyrhynchus*. Presented estimates are coefficients from a model with “Year” and “Bird ID” as random effects and “Day of month”, “Sex”, “Supplementary feeding”, “Sex * Supplementary feeding” and “Year * Supplementary feeding” as fixed effects. The supplementary feeding variable distinguishes between birds with API assessments inside and outside areas with supplementary feeding, and N indicates the number of geese included in the analysis. Day of month and Year was fitted as continuous variables (covariates).

N = 6947	Estimate	SE	P value
Day of month	0.060	0.001	<0.001*
Sex	0.146	0.021	<0.001*
Supplementary feeding	0.001	0.012	0.939
Sex * Supplementary feeding	-0.017	0.012	0.146
Year * Supplementary feeding	0.002	0.002	0.444

* Significant effects on α-level 0.05

## Discussion

Negative impacts of harsh winters are often reported in studies of migratory waterbirds [13,31,32], but the persistence of these effects in subsequent seasons is rarely investigated. From continuous assessments of pink-footed geese body condition during the entire spring season, this study supports the three hypotheses that 1) average early spring body condition is affected by the harshness of the preceding winter, 2) on a population level pink-footed geese are capable of compensating the lower energetic starting point during the course of spring-fattening and 3) spring-fattening rates of individual geese are state-dependent and inversely proportional to early spring body condition. Our data on spring-fattening rates of individual geese showed that birds entering spring in poor condition accumulated body mass to a greater extent than birds in good condition, but could not fully compensate the lower starting point of spring-fattening. The inability to fully compensate a poor body condition was consistent irrespective of winter harshness, and as average late spring body condition of the entire population did not differ between mild and cold winters, the individual variation in body condition persisting throughout spring was probably related to differences in life-history events other than winter harshness. Individual variation in early spring body condition may be driven by differences in dominance rank, family status, migration history, site use, weather impacts, age, illness and disturbance levels during the period leading up to spring. Many of these impacts may persist on longer temporal scales and carry over to the spring period. Collectively these factors may lead to substantial differences in late spring body condition among years [33], although the effect of the preceding winter is no longer discernible.

Collectively our results indicate that 1) the effect of a harsh winter does not persist long enough to influence late spring body condition and subsequent reproduction [28], but 2) other life history-factors may lead to a carry-over effect of early spring body condition of individual geese, suggesting that variation in the amount of energy reserves carried in to spring is very important. We interpret this as indicating that although the effect of winter harshness can be offset during the course of spring-fattening, different life histories might affect the relative position of individual geese on the entire spectrum of body conditions. This is in good agreement with previous findings suggesting that some individuals in goose populations fare consistently better than others [34]. While it seems somewhat contradictory that a partly compensation of individual body condition during spring is enough to ensure full compensation of winter effects on the population level, this is a consequence of the fact that individual variation is driven by many other important life-history events than winter temperature.

Females were compensating the lower energetic starting point to a greater extent than males, which may relate to sex-specific differences in spring behaviour and spring target mass. While paired males spend proportionately more time on aggressive interactions and vigilance, females forage more intensively to prepare for the forthcoming breeding event [35,36].

The fading effect of winter harshness on average body condition and inverse proportionality between spring-fattening rates and early spring body condition both suggest a state-dependent physiological mechanism among spring-fattening geese, allowing them to regulate energy intake (e.g. time spend foraging) based on current energetic state. Hence, individuals entering spring in poor body condition might speed up energy accumulation, individuals entering spring in good condition may hold back, or the result may be a combination of both. Although this indicates that pink-footed geese have a buffer for building up body mass during spring-fattening, the maximum late spring body condition that individuals can reach still seemed to be restricted by their body condition upon entering spring. Spring-fattening is generally described as a very busy time among many species of geese, but recent studies now suggest that during early phases of spring migration geese may accumulate energy stores below their maximum capacity [37,38]. Pink-footed geese might restrict the build-up of energy stores until later in spring in order not to pay the additional costs (locomotory, predator avoidance etc.) of a larger body mass [39,40]. State-dependent foraging has been demonstrated in other bird species on short temporal scales [41,42], but this study emphasizes that a similar mechanism may regulate foraging decisions on a seasonal basis. Higher fat deposition rates for geese in poor spring condition have also been shown for barnacle geese (*Branta leucopsis*) during a three weeks stopover in Norway [43]. Like in our analysis, this study also found that lean individuals were unable to fully catch up with birds arriving at this stopover in better condition. Collectively these findings indicate that although energy accumulation may operate as a state-dependent mechanism, it is not always enough to compensate for lost opportunities in the past.

Based on current data we found no support for an effect of supplementary feeding in April, which seemed to confirm hypothesis 4. One possible explanation of this could be that food is plentiful during spring, and that geese are already capable of foraging at acceptable intake rates in natural habitats. As such, the build-up of energy stores seemed not to be limited by food availability during this period. The fact that geese exploiting supplementary feeding sites did not boost their body condition supports the state-dependent foraging hypothesis during spring-fattening (hypothesis 3). In the current situation supplementary feeding seems to alleviate conflicts with agriculture without any greater effect on goose body condition.

Recent studies have highlighted the importance of temporal scale when studying environmental impacts [44,45], and an increasing number of papers conclude that waterbirds are able to partly compensate past processes in a subsequent period [44–46]. Even in systems where carry-over effects are known to drive fitness asymmetries, the implications of these are strongly dependent on fitness determinants in subsequent seasons [46,47]. Changing environmental conditions are manifold, and potential long-term impacts and individual carry-over effects may concern any part of past life histories. Site use [48], hunting exposure [49], disturbance [50], parental status [51], captivity [52] and winter harshness (this study) are among studied examples on geese. What all these have in common is the dependence on temporal scale, as the impact of most effects are likely to fade with time. The real question therefore is whether the impact persists long enough to affect a future life-history event influencing important demographic traits such as reproduction and survival. In this study of winter harshness and pink-footed geese this does not seem to be the case, as we found no indications of a population level effect of winter on pre-breeding body mass which is a known proxy of future reproduction [28].

Besides the reliance on temporal scale, this study also highlights the importance of population vs individual effects. While the population of pink-footed geese was able to fully compensate the effect of harsh winters during spring-fattening, individual differences in energy stores when entering spring persisted partly throughout the spring period. Although the effect of winter may only be short-term, individual variation in early spring body condition related to the life history of individual birds might to some extent carry over to subsequent seasons and potentially influence fitness further ahead [9]. This might indicate a carry-over effect on the individual level that could relate to differences in dominance rank, site use, disturbance, migratory strategy etc.

## Supporting Information

S1 Table Average winter temperatures in the wintering areas of pink-footed geese *Anser brachyrhynchus* and no. of API assessments throughout the monitoring period. (PDF)

S2 Table Ringing data for birds included in the current study. (XLSX)

S3 Table Re-sightings and API assessments of birds included in the current study. (XLSX)

## References

[1] Forsman JT, Monkkonen M (2003) The role of climate in limiting European resident bird populations. J Biogeogr 30: 55–70.

[2] Andrewartha HG, Birch LC (1984) The ecological web: more on the distribution and abundance of animals Chicago: University of Chicago Press. 506 p.

[3] Paul MJ, Zucker I, Schwartz WJ (2008) Tracking the seasons: the internal calendars of vertebrates. Philos T R Soc B 363: 341–361. 10.1098/rstb.2007.2143 PMC2606754 17686736

[4] Alerstam T, Hedenström A, Åkesson S (2003) Long-distance migration: evolution and determinants. Oikos 103: 247–260.

[5] Gray DR (1993) Behavioral Adaptations to Arctic Winter—Shelter Seeking by Arctic Hare (Lepus-Arcticus). Arctic 46: 340–453.

[6] Loison A, Langvatn R (1998) Short- and long-term effects of winter and spring weather on growth and survival of red deer in Norway. Oecologia 116: 489–500. 28307518 10.1007/s004420050614

[7] Clausen P, Frederiksen M, Percival SM, Anderson GQA, Denny MJH (2001) Seasonal and annual survival of East-Atlantic Pale-bellied Brent Geese Branta hrota assessed by capture-recapture analysis. Ardea 89: 101–111.

[8] Saino N, Szep T, Ambrosini R, Romano M, Moller AP (2004) Ecological conditions during winter affect sexual selection and breeding in a migratory bird. P Roy Soc B-Biol Sci 271: 681–686. 10.1098/rspb.2003.2656 PMC1691647 15209100

[9] Guillemain M, Elmberg J, Arzel C, Johnson AR, Simon G (2008) The income-capital breeding dichotomy revisited: late winter body condition is related to breeding success in an income breeder. Ibis 150: 172–176.

[10] Gauthier G, Pradel R, Menu S, Lebreton JD (2001) Seasonal survival of Greater Snow Geese and effect of hunting under dependence in sighting probability. Ecology 82: 3105–3119.

[11] Madsen J, Frederiksen M, Ganter B (2002) Trends in annual and seasonal survival of Pink-footed Geese Anser brachyrhynchus. Ibis 144: 218–226.

[12] Owen M, Wells RL, Black JM (1992) Energy Budgets of Wintering Barnacle Geese—the Effects of Declining Food Resources. Ornis Scand 23: 451–458.

[13] Raveling DG (1979) The Annual Cycle of Body Composition of Canada Geese with Special Reference to Control of Reproduction. Auk 96: 234–252.

[14] Madsen J, Tjørnløv RS, Frederiksen M, Mitchell C, Sigfusson AT (2014) Connectivity between flyway populations of waterbirds: assessment of rates of exchange, their causes and consequences. J Appl Ecol 51: 183–193.

[15] Houston AI, McNamara JM (1999) Models of Adaptive Behaviour: An Approach Based on State. Cambridge, England: Cambridge University Press. 390 p.

[16] Norris DR (2005) Carry-over effects and habitat quality in migratory populations. Oikos 109: 178–186

[17] Norris DR, Marra PP (2007) Seasonal interactions, habitat quality, and population dynamics in migratory birds. Condor 109: 535–547.

[18] Hawley DM, DuRant SE, Wilson AF, Adelman JS, Hopkins WA (2012) Additive metabolic costs of thermoregulation and pathogen infection. Funct Ecol 26: 701–710.

[19] Drewien RC, Bouffard SH (1994) Winter body mass and measurements of Trumpeter Swans Cygnus buccinator. Wildfowl 45: 22–32.

[20] Madsen J (2008) Fodring af kortnæbbede gæs om foråret i Vestjylland. Biologiske fakta til understøttelse af fremtidig forvaltningsstrategi National Environmental Research Institute, Aarhus University. 20 p.

[21] Madsen J (1996) Exposure of spring-staging pink-footed geese Anser brachyrhynchus to pesticide-treated seed. Wildlife Biol 2: 1–9.

[22] Madsen J (1984) Numbers, distribution and habitat utilization of pink-footed geese in Denmark 1980–1983. Norsk Polarinstitutt Skrifter 181: 19–23.

[23] Fox AD, Madsen J, Boyd H, Kuijken E, Norriss DW, Tombre IM, et al (2005) Effects of agricultural change on abundance, fitness components and distribution of two arctic-nesting goose populations. Global Change Biol 11: 881–893.

[24] Madsen J, Cracknell G, Fox AD, editors (1999) Goose populations of the western Palearctic. A review of status and distribution. Wetlands International, Wageningen, The Netherlands, National Environmental research institute, Rönde, Denmark: Wetlands International Publ. No. 48. 344 p.

[25] Owen M (1981) Abdominal Profile—a Condition Index for Wild Geese in the Field. J Wildlife Manage 45: 227–230.

[26] Madsen J, Klaassen M (2006) Assessing body condition and energy budget components by scoring abdominal profiles in free-ranging pink-footed geese Anser brachyrhynchus. J Avian Biol 37: 283–287.

[27] Feret M, Bety J, Gauthier G, Giroux JF, Picard G (2005) Are abdominal profiles useful to assess body condition of spring staging Greater Snow Geese? Condor 107: 694–702.

[28] Drent R, Both C, Green M, Madsen J, Piersma T (2003) Pay-offs and penalties of competing migratory schedules. Oikos 103: 274–292.

[29] Clausen KK, Madsen J (2014) Effects of neckbands on body condition of migratory geese. J Ornithol 155: 951–958.

[30] R Development Core Team (2008) R: A language and environment for statistical computing. Vienna, Austria: R Foundation for Statistical Computing.

[31] Sedinger JS, Ward DH, Schamber JL, Butler WI, Eldridge WD, Conant B, et al (2006) Effects of El Nino on distribution and reproductive performance of Black Brant. Ecology 87: 151–159. 16634306 10.1890/04-1013

[32] Robb JR, Tori GM, Kroll RW (2001) Condition indices of live-trapped American black ducks and mallards. J Wildlife Manage 65: 755–764.

[33] Alisauskas RT (2002) Arctic climate, spring nutrition, and recruitment in mid-continent lesser snow geese. J Wildlife Manage 66: 181–193.

[34] Owen M, Black JM (1989) Barnacle Goose In: Newton I (Ed.) Lifetime reproduction in Birds, pp. 349–362. Academic Press, London.

[35] Teunissen W, Spaans B, Drent R (1985) Breeding Success in Brent in Relation to Individual Feeding Opportunities during Spring Staging in the Wadden Sea. Ardea 73: 109–119.

[36] Black JM, Owen M (1989) Agonistic Behavior in Barnacle Goose Flocks—Assessment, Investment and Reproductive Success. Anim Behav 37: 199–209.

[37] Bauer S, Madsen J, Klaassen M (2006) Intake rates, stochasticity, or onset of spring—what aspects of food availability affect spring migration patterns in Pink-footed Geese Anser brachyrhynchus? Ardea 94: 555–566.

[38] Ely CR (1992) Time Allocation by Greater White-Fronted Geese—Influence of Diet, Energy Reserves and Predation. Condor 94: 857–870.

[39] Houston AI, McNamara JM, Hutchinson JMC (1993) General Results Concerning the Trade-Off between Gaining Energy and Avoiding Predation. Philos T Roy Soc B 341: 375–397.

[40] Rogers CM, Smith JNM (1993) Life-History Theory in the Nonbreeding Period—Trade-Offs in Avian Fat Reserves. Ecology 74: 419–426.

[41] Lilliendahl K, Carlson A, Welander J, Ekman JB (1996) Behavioural control of daily fattening in great tits (Parus major). Can J Zool 74: 1612–1616.

[42] Ekman JB, Hake MK (1990) Monitoring starvation risk: adjustments of body reserves in greenfinches (Carduelis chloris L.) during periods of unpredictable foraging success. Behav Ecol 1: 62–67.

[43] Prop J, Black JM, Shimmings P (2003) Travel schedules to the high arctic: barnacle geese trade-off the timing of migration with accumulation of fat deposits. Oikos 103: 403–414.

[44] Conklin JR, Battley PF (2012) Carry-over effects and compensation: late arrival on non-breeding grounds affects wing moult but not plumage or schedules of departing bar-tailed godwits Limosa lapponica baueri. J Avian Biol 43: 252–263.

[45] Senner NR, Hochachka WM, Fox JW, Afanasyev V (2014) An Exception to the Rule: Carry-Over Effects Do Not Accumulate in a Long-Distance Migratory Bird. PLoS ONE 9: e86588 10.1371/journal.pone.0086588 24523862 PMC3921144

[46] Ockendon N, Leech D, Pearce-Higgins JW (2013) Climatic effects on breeding grounds are more important drivers of breeding phenology in migrant birds than carry-over effects from wintering grounds. Biol Lett 9: 20130669 10.1098/rsbl.2013.0669 24196517 PMC3871353

[47] Harrison XA, Hodgson DJ, Inger R, Colhoun K, Gudmundsson GA, McElwaine G, et al (2013) Environmental Conditions during Breeding Modify the Strength of Mass-Dependent Carry-Over Effects in a Migratory Bird. PLoS ONE 8: e77783 10.1371/journal.pone.0077783 24143258 PMC3797109

[48] Schamber JL, Sedinger JS, Ward DH (2012) Carry-over Effects of Winter Location Contribute to Variation in Timing of Nest Initiation and Clutch Size in Black Brant (Branta Bernicla Nigricans). Auk 129: 205–210.

[49] Juillet C, Choquet R, Gauthier G, Lefebvre J, Pradel R (2012) Carry-over effects of spring hunt and climate on recruitment to the natal colony in a migratory species. J Appl Ecol 49: 1237–1246.

[50] Morrissette M, Bety J, Gauthier G, Reed A, Lefebvre J (2010) Climate, trophic interactions, density dependence and carry-over effects on the population productivity of a migratory Arctic herbivorous bird. Oikos 119: 1181–1191.

[51] Inger R, Harrison XA, Ruxton GD, Newton J, Colhoun K, Gudmundsson GA, et al (2010) Carry-over effects reveal reproductive costs in a long-distance migrant. J Anim Ecol 79: 974–982. 10.1111/j.1365-2656.2010.01712.x 20579179

[52] Legagneux P, Fast PLF, Gauthier G, Bety J (2012) Manipulating individual state during migration provides evidence for carry-over effects modulated by environmental conditions. P Roy Soc B-Biol Sci 279: 876–883. 10.1098/rspb.2011.1351 PMC3259927 21865256

