# Supplementary material for: Carry-Over or Compensation? The Impact of Winter Harshness and Post-Winter Body Condition on Spring-Fattening in a Migratory Goose Species
Source: PLoS One. 2015 Jul 2;10(7):e0132312. doi: 10.1371/journal.pone.0132312 (PMC4489791; doi:10.1371/journal.pone.0132312)
Supplement: S1 Table — (PDF) [file pone.0132312.s001.pdf]

Supporting information

Appendix S1: Average winter temperatures in the wintering areas of pink-footed geese *Anser brachyrhynchus* and no. of API assessments throughout the monitoring period.

Year	Average winter temperature[a]	No. of API assessments (adult birds excl. year of ringing)
1991	2.11	309
1992	4.04	694
1993	3.42	2643
1994	3.42	3162
1995	4.71	1590
1996	-0.69	2867
1997	1.41	1133
1998	4.64	112
1999	3.81	169
2000	4.63	506
2001	3.44	651
2002	4.25	484
2003	1.77	707
2004	3.82	1108
2005	3.89	475
2006	2.55	1825
2007	6.28	1271
2008	4.77	96
2009	2.10	1064
2010	0.77	831
2011	1.24	986
2012	3.47	916
2013	1.95	910

[a] Composite average covering the main winter staging areas of pink-footed geese: Esbjerg (Denmark), Leeuwarden (The Netherlands) and Oostende (Belgium).
